# Enhanced Sorption of Europium and Scandium Ions from Nitrate Solutions by Remotely Activated Ion Exchangers

**DOI:** 10.3390/polym15051194

**Published:** 2023-02-27

**Authors:** Aldan Imangazy, Talkybek Jumadilov, Khuangul Khimersen, Arman Bayshibekov

**Affiliations:** 1 Laboratory of Synthesis and Physicochemistry of Polymers, Bekturov Institute of Chemical Sciences, 106 Sh. Ualikhanov Str., Almaty 050010, Kazakhstan; 2 School of Chemical Engineering, Kazakh-British Technical University, 59 Tole bi Street., Almaty 050000, Kazakhstan; 3 Institute of Natural Sciences and Geography, Abai Kazakh National Pedagogical University, 13 Dostyk Ave., Almaty 050010, Kazakhstan

**Keywords:** ion exchangers, interpolymer system, remote interaction effect, europium ions, scandium ions, sorption

## Abstract

The escalating demand for rare earth metals (REM) in situations of limited availability has spurred scientists to seek alternative sources of REM, such as industrial waste solutions. This paper investigates the potential for improving the sorption activity of readily available and inexpensive ion exchangers, specifically the interpolymer systems “Lewatit CNP LF and AV-17-8”, towards europium and scandium ions, in comparison to the unactivated ion exchangers. The sorption properties of the improved sorbents (interpolymer systems) were evaluated using conductometry, gravimetry, and atomic emission analysis. The results demonstrate that the “Lewatit CNP LF:AV-17-8” (5:1) interpolymer system exhibits a 25% increase in europium ion sorption compared to the raw Lewatit CNP LF (6:0), and a 57% increase in europium ion sorption compared to the raw AV-17-8 (0:6) ion exchanger after 48 h of the sorption process. In contrast, the “Lewatit CNP LF:AV-17-8” (2:4) interpolymer system exhibits a 310% increase in scandium ion sorption compared to the raw Lewatit CNP LF (6:0), and a 240% increase in scandium ion sorption compared to the raw AV-17-8 (0:6) after 48 h of interaction. The improvement in europium and scandium ion sorption levels by the interpolymer systems, compared to the raw ion exchangers, may be attributed to the high ionization degree resulting from the remote interaction effect of the polymer sorbents as the interpolymer system in aqueous media.

## 1. Introduction

Rare earth metals (REM) have recently gained an essential role in high-tech applications. They are crucial components in a wide range of modern technologies, from smartphones and electric vehicles to wind turbines and medical devices [1,2,3,4]. The worldwide REM market is influenced by a number of factors, including global demand, supply, and the price of metals [5]. These metals have unique properties that make them essential for many high-tech applications, such as their ability to conduct electricity and retain magnetic fields [6]. The importance of rare earth metals lies in their unique properties and their irreplaceability in many applications. For example, europium and scandium are two chemical elements that have significant importance in a variety of industrial and technological applications [7,8].

Europium is a silvery-white color soft strategic metal, belonging to the cerium group of rare-earth light lanthanides family [9]. It is a key component in the production of phosphors, which are used in fluorescent lamps and television screens by mixing with other rare-earth elements to produce bright and vibrant colors [10]. Additionally, europium is used in nuclear reactors as a control rod material to regulate the rate of nuclear reactions [11]. In medicine, europium can be used as a contrast agent for magnetic resonance imaging (MRI) and computed tomography (CT) scans due to its magnetic properties [12]. The supply of europium is limited, with most of the world’s production coming from China, which produces around 85% of the world’s supply [13]. Other producers of europium include Brazil, the United States, and Australia [14]. The limited supply of europium and the concentration of production in China have led to concerns about the availability of the metal and the potential for price volatility [15].

Scandium is a rare earth metal that has attracted attention due to its unique properties and potential applications in various industries [16,17,18,19]. The worldwide scandium market has seen significant growth in recent years [20], driven by increasing demand from the aerospace, defense, and energy sectors. In the aerospace industry, scandium is used to improve the strength and durability of aluminum alloys, which are widely used in aircraft manufacturing [21,22]. The addition of small amounts of scandium to aluminum alloys can significantly improve their mechanical properties, making them more resistant to fatigue and corrosion [23]. This has led to increased demand for scandium in the aerospace industry, with major players like Airbus, Boeing, and Bombardier investing in research and development to incorporate scandium into their aircraft [24]. Additionally, these metal compounds are widely used as catalysts in the chemical industry [25,26,27]. The defense sector is another important market for scandium which is used to make high-strength, lightweight materials for military applications such as armor plating and missile guidance systems [28]. In addition to its use in aerospace and defense, scandium has potential applications in the energy sector. Scandium is a key component in solid oxide fuel cells (SOFCs), which are an emerging technology for clean energy generation [29]. SOFCs have the potential to replace traditional power generation methods, such as combustion-based systems, and offer higher energy efficiency and lower emissions. The use of scandium in SOFCs has been shown to improve their performance and reduce their cost, making them more competitive with other energy sources.

The worldwide europium and scandium market is still in its early stages, and there are currently only a handful of producers. The majority of REMs is produced as a byproduct of other mining operations. The demand for europium and scandium is growing annually, but their availability on Earth is limited [30]. However, most mining enterprises generate trace amounts of europium and scandium in their process fluids as a co-product, and their recovery could provide an additional supply of these rare earth elements [31,32].

For the recovery of REMs from various aqueous solutions, many treatment processes have been used, including adsorption [33], membrane separation [34,35], precipitation [36], filtration [37], ion exchange [38], solvent extraction [39], and electrochemical treatment [40]. However, the above-mentioned technologies have their advantages and disadvantages. For example, membrane separation and ion exchange techniques are expensive while processing large volumes of liquids containing low concentrations of metal ions. As a result, these technologies are not economically feasible for small-scale companies. Another technology, solvent extraction, is also costly, complicated, and time-consuming, as well as requiring a huge volume of solvent, but has efficiency and selectivity. Other REM ion recovery technologies such as precipitation and electrochemical treatment methods are simple to apply but they are not efficient when the metal ions are present in trace amounts [41]. As a result, the sorption methods are technologically preferred due to many advantages (more environmentally friendly and having fewer technological cycles) as opposed to other extraction technologies [42]. For the production of valuable components from solutions, adsorption processes might use such materials as specific polymers, activated carbons, biological sorbents, etc., which have recently grown interesting [43,44,45,46,47].

An effective method for europium and scandium ions sorption out of solutions might become the use of commercially low-cost ion exchangers, for instance, Lewatit CNP LF and AV-17-8. The Lewatit CNP LF ion exchanger is mostly used for the decarbonization and softening of drinking water [48], whereas the AV-17-8 ionite basically is designed for wastewater treatment and sorption of non-ferrous metals [49]. For this reason, this paper investigates the use of Lewatit CNP LF and AV-17-8 ionites for europium and scandium ions sorption from nitrate solutions. Based on our previous study [50], for this research, we decided to choose Lewatit CNP LF and AV-17-8 ion exchangers due to their availability and low cost. The combinations of ion exchangers in the interpolymer system “Lewatit CNP LF:AV-17-8” (X:Y, molar ratios) can be used to examine the impact of remote interaction on enhancing the degree of europium and scandium ions sorption. For clarification, an interpolymer system is a system of two cross-linked polymers of acidic and basic nature, with active functional groups which are in a common solution but without direct contact. As a result of the mutual activation during their remote interaction in aqueous media, the polymers obtain a highly ionized state [51,52], which results in a significant increase in the degree of metal ions sorption by the interpolymer system in comparison with the raw polymers. In this regard, the goal of this research is to investigate the sorption activity towards europium and scandium ions of activated Lewatit CNP LF and AV-17-8 ion exchangers in the interpolymer system “Lewatit CNP LF:AV-17-8” (X:Y) from nitrate solutions.

## 2. Materials and Methods

### 2.1. Materials

For this research, we used the following polymeric materials: (1) Lewatit CNP LF (H^+^ form) (LANXESS Deutschland GmbH, Cologne, Germany), a weakly acidic macroporous cation exchanger (crosslinked polyacrylate with granule size 0.3–1.6 mm), and (2) AV-17-8 (OH^−^ form) (Azot, Cherkasy, Ukraine), a strongly basic anion exchanger (a copolymer of styrene and divinylbenzene with granule size 0.3–1.2 mm).

For our experiments, the following reagents were used: europium(III) nitrate pentahydrate (Sigma-Aldrich, Darmstadt, Germany) as a source of europium ions in the solution; scandium(III) nitrate hydrate (Sigma-Aldrich, Darmstadt, Germany) as a source of scandium ions in the solution; arsenazo III-metal indicator reagent (Sigma-Aldrich, Germany) as a color-forming reagent to prepare detectable forms of the europium and scandium ions complexes in the samples; perchloric acid (HClO_4_, ACS reagent, 70%) (Sigma-Aldrich, Darmstadt, Germany) as a reagent for the preparation of the standard solution. 

The following measuring instruments and equipment were used: pH-meter (conductometer PC 50 VioLab Complete Kit (XS Instruments, Carpi, Italy) for the measurements of the hydrogen ions concentration (to study the acid-base properties of the solution) and the specific conductance of the solutions (to characterize the equilibrium of polyelectrolytes dissociation and comprehend ion charge transfer). The mass of the materials was determined using an analytical balance SHIMADZU AY220 (Shimadzu Corporation, Kyoto, Japan). A Jenway-6305 spectrophotometer (Cole-Parmer, Jenway, York, UK) was used to measure the optical density of the solutions in order to calculate the concentrations of europium(III) and scandium(III) ions. The Optima 8300DV Duo inductively coupled plasma-optical emission spectrometer (ICP-OES) (PerkinElmer, Waltham, MA, USA) with a wavelength range of 165–782 nm was used to identify the residual ions of europium(III) and scandium(III) in the samples. FTIR spectra for tested ion exchangers were provided by NICOLET 5700 spectrophotometer (Thermo Fischer Scientific, Waltham, MA, USA). The measurement errors were less than 1%.

### 2.2. Preparation of the Interpolymer System

The first step in preparing an interpolymer system is to select the polymers that will be used. These polymers should have compatible chemical properties, so they could remotely interact with each other in an aqueous medium due to the different natures of polymers (acidic/basic). Our previous studies have demonstrated that the interpolymer system is a pair of crosslinked polymers with active functional groups which are in a common solution but without mutual contact [42,53]. In the present research, the weakly acidic and strongly basic components of the interpolymer system were Lewatit CNP LF (H^+^ form) and AV-17-8 (OH^−^ form) ion exchangers. These ion exchangers Lewatit CNP LF (X) and AV-17-8 (Y) were weighted and put in polypropylene meshes according to their X:Y molar ratios. The interpolymer system “Lewatit CNP LF:AV-17-8” with molar ratios X:Y of 6:0, 5:1, 4:2, 3:3, 2:4, 1:5, and 0:6 were prepared for further research.

### 2.3. Polymers Swelling Coefficient Determination

**Figure 1:**
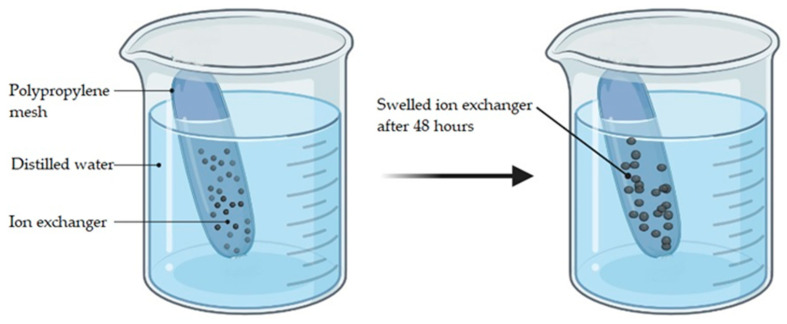
Representation of swelling process of ion exchangers for polymers swelling coefficient determination.

During swelling, dissociation of ionogenic groups of ion exchangers occurs, negative charge accumulates on the macromolecule which then repels, and the size of the polymer tangle increases to a certain value. For our experiments, the ion exchangers Lewatit CNP LF (X) and AV-17-8 (Y) have been put apart in polypropylene meshes according to X:Y molar ratios of 6:0, 5:1, 4:2, 3:3, 2:4, 1:5, and 0:6, and placed in distilled water for 48 h for swelling (Figure 1).

The gravimetric method is a widely used technique for estimating swelling coefficients. This coefficient is typically expressed as a ratio of the change in volume to the original volume. In this study, the gravimetric method was used to estimate the swelling coefficients (*K_sw_*) of the ion exchangers. The dry and swollen polymers were weighed, and *K_sw_* was calculated using the following Equation (1):

(1) $$K_{sw}=\frac{m_{2}-m_{1}}{m_{1}}$$

where *m*_1_ and *m*_2_ (in g) are masses of the dry and swelled polymers, respectively. 

The swelling coefficient represents the change in the volume of an ion exchanger when it adsorbs a certain amount of fluid. The gravimetric method is often preferred due to its simplicity and ease of use, as well as its ability to provide accurate and reliable results.

### 2.4. Mutual Activation of the Interpolymer System

**Figure 2:**
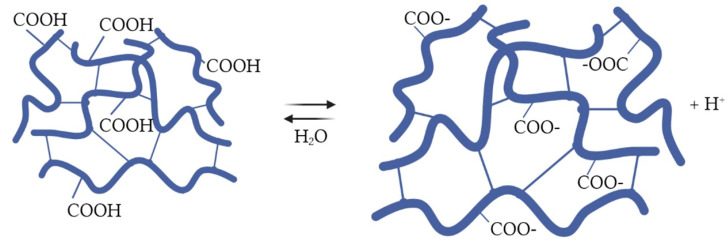
The dissociation of Lewatit CNP LF ion exchanger in aqueous solution.

**Figure 3:**
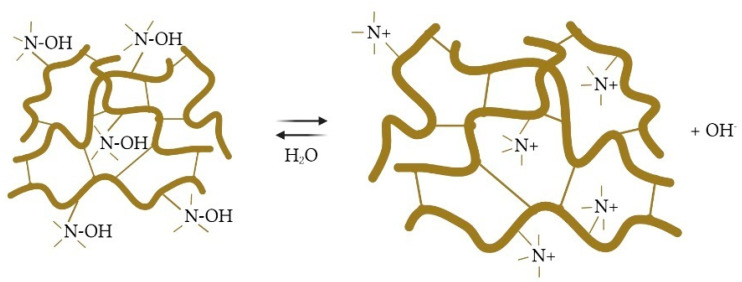
The dissociation of AV-17-8 ion exchanger in aqueous solution.

In aqueous solutions, ion exchangers can either release or accept protons depending on their chemical structure and the surrounding pH. This behavior is due to the presence of functional groups on the ion exchanger’s surface, which can either donate or accept protons. The functional groups are typically either acidic or basic, and their dissociation can be described by an acid-base equilibrium. In aqueous media, ion exchangers dissociate through a series of steps. The first step involves the attraction of the ion to the functional group on the ion exchanger. This is followed by a second step, in which the ion is exchanged with a counter ion that is already bound to the ion exchanger. This counter ion is released into the solution [54]. According to [55], the acid-base properties of ion exchangers are associated with their dissociation in an aqueous media. Several factors can influence the dissociation of ion exchangers in aqueous media, including pH, temperature, and the concentration of the ions in the solution. There are two main dissociation steps of studied ion exchangers that occurred in the aqueous solution:

1. Dissociation of Lewatit CNP LF in an aqueous solution occurs according to Figure 2.

2. Dissociation of AV-17-8 in an aqueous solution occurs according to Figure 3.

**Figure 4:**
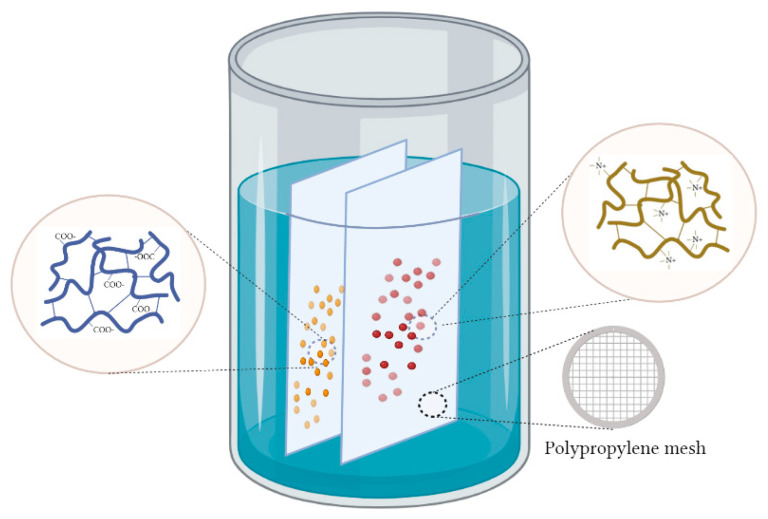
Illustration of the activation process of the “Lewatit CNP LF:AV-17-8” (X:Y) interpolymer system by remote interaction in an aqueous solution.

The activation of the interpolymer system is necessary to shift the ion exchangers into a highly ionized state by modifying their conformational and electrochemical characteristics through remote interaction [50] (p.3). For this, the polypropylene meshes with swelling ion exchangers inside (Figure 4) were inserted at a distance of about 1–2 cm opposite each other in a glass with distilled water, establishing the interpolymer system “Lewatit CNP LF:AV-17-8” in a certain molar ratio (X:Y) (Figure 4). It should be mentioned that polypropylene meshes are widely used in various industrial and biomedical applications due to their unique properties, such as excellent chemical resistance, mechanical strength, and biocompatibility. One important aspect of their performance is their neutrality to aqueous media. Polypropylene is a non-polar thermoplastic polymer that is hydrophobic in nature, meaning it repels water. When polypropylene meshes are exposed to the studied aqueous environment, they remain chemically and physically stable due to their low solubility and excellent resistance to water [56].

The mechanism of the remote interaction effect implies the complete exclusion of the direct interaction of acidic and basic polymers due to the constantly changing morphology of the surface. For the explanation of this effect, in our case, we can consider the dissociation of the ion exchangers Lewatit CNP LF (H^+^ form) and AV-17-8 (OH^−^ form) by the formation of free H^+^ and OH^−^ ions, respectively. Then, released H^+^ and OH^−^ ions form weakly dissociated water molecules and leave the functional groups of polyelectrolytes activated and stabilized by intramolecular interactions. Moreover, according to Pearson’s hard and soft acid and bases (HSAB) theory [57], which states that “hard acids prefer to bond to hard bases, and soft acids prefer to bond to soft bases” [58], released hard acid H^+^ (Figure 2) and hard base OH^−^ (Figure 3) similarly form water molecules and leave the functional groups of ion exchangers stabilized and activated by intramolecular interactions. As a result, concentrations of oxonium (H_3_O^+^) and OH^−^ ions became significantly higher around the Lewatit CNP LF and AV-17-8, respectively. This gradient presumably makes the concentrations of neutral water around the ion exchangers in this interpolymer system lower than the concentrations around the singly used ion exchangers, and enhances the dissociation of counter ions from the ionic groups in the polyelectrolytes [32] (p.3). The result of the remote interaction is the mutual activation of the initial ion exchangers Lewatit CNP LF and AV-17-8 in the interpolymer pair and, as a consequence, the transition of their macromolecules to a highly ionized state, which further leads to a significant increase in the sorption properties towards the europium and scandium ions of polyacids and polybases in the interpolymer system “Lewatit CNP LF:AV-17-8” (X:Y).

### 2.5. Polymer Chain Binding Degree Determination

The polymer chain binding degree (*θ*) specifies the number of units around the central metal ion and directly depends on the ionization degree of the ion exchangers during their remote interaction in the interpolymer system “Lewatit CNP LF:AV-17-8” (X:Y). This parameter (*θ*) was calculated according to Equation (2) [32] (p.3):

(2) $$\theta =\frac{\vartheta_{sorbed}}{\vartheta_{is}}\times 100\;\%$$

where the amount of *ϑ_sorbed_* is the amount of sorbed europium and scandium ions (in mol); *ϑ_is_* is the amount (in mol) of the interpolymer system “Amberlite IR120:AV-17-8 (X:Y).

### 2.6. Plotting a Calibration Curve

The method for europium and scandium ions determination is based on the formation of a colored complex compound of an organic analytical reagent arsenazo III with europium and scandium ions. To get an analytical form, it was required to introduce a colored reagent such as arsenazo III, which is a bisazo-derivative of chromotropic acid [59].

The optical density (D) values of the formed europium and scandium ions complexes in the solutions were determined by the “KFK-3M” spectrophotometer. The calibration curve was plotted using the “Origin” software (r^2^ value was 0.99605, D = 0.0655C − 0.0245).

### 2.7. Determination of Residual Concentration of Europium and Scandium Ions

For the experiments, separate 1100 mL of the europium(III) and scandium(III) nitrate solutions with concentration C = 300 mg/L was poured into 11 glasses (100 mL each). The Lewatit CNP LF and AV-17-8 ionites were put separately in 2 polypropylene meshes in common glass with a solution in accordance with their molar ratios X:Y to form the interpolymer system “Lewatit CNP LF:AV-17-8” (X:Y). For spectrophotometric analysis, one aliquot (1 mL) was taken from each solution at the set time. The aliquot sampling time was 0.08, 1, 2, 6, 24, and 48 h. In the final step, 99 aliquots of solutions were obtained.

For spectrophotometric analysis, each aliquot (1 mL) with an unknown concentration of the analyte was transferred into the volumetric flasks (50 mL). Then, 12 mL of arsenazo (0.015%) and 2 mL of perchloric acid solution (0.08 M) were poured into each flask. After that, the volume of each solution was brought to 50 mL with distilled water, and after 15 min, the measurements were started. The reference solution contained all of the above components except the analyte.

**Figure 5:**
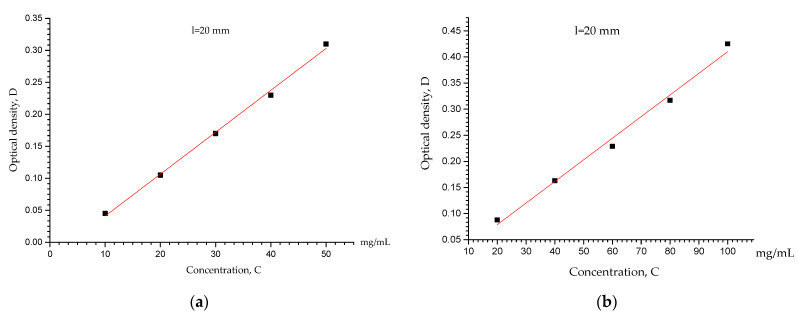
The calibration curves for determining the (**a**) europium(III) and (**b**) scandium(III) concentrations in the test solutions.

The optical density data for the tested solutions with unknown europium and scandium ion concentrations were reported. We found the unknown concentration corresponding to each signal after obtaining the value of the analytical signal (D) and utilizing the provided calibration curves (Figure 5). The optical density (D) of solutions containing europium and scandium ions was measured using a Jenway-6305 spectrophotometer in order to calculate the concentrations of europium(III) and scandium(III) ions. Each measurement was taken three times. The sorption degree was calculated using the following Equation (3) [32] (p.4):

(3) $$\eta =\frac{C_{initial}-C_{residual}}{C_{initial}}\times 100\%$$

where *C_initial_* and *C_residual_* are the initial and residual concentrations (in g/L) of europium and scandium ions in solutions, respectively.

The sorption degree of a polymer can be used to optimize its performance in a particular application. For example, by understanding the sorption properties of a polymer, it may be possible to modify its surface chemistry to increase its selectivity for a particular substance or to enhance its stability under specific environmental conditions.

### 2.8. ICP-OES Analysis

ICP-OES (*Inductively Coupled Plasma Optical Emission Spectroscopy*) as a powerful analytical technique was used to detect and quantify europium and scandium elements in a sample. ICP-OES worked by converting a sample into plasma by heating it. The plasma then was excited by radiofrequency energy and emits light that was analyzed by a spectrometer. The spectral lines emitted by the plasma correspond to the elements present in the sample. The intensity of each spectral line was proportional to the concentration of the corresponding element (europium or scandium) in the sample. By measuring the intensity of the spectral lines, the elemental composition of the sample was determined. The graphical representations of the findings are presented in the Results and Discussion section.

### 2.9. FTIR Spectroscopy

FTIR (Fourier Transform Infrared) spectroscopy as a widely used analytical technique in chemistry and materials science was used to measure the absorption (or transmission) of infrared radiation by a sample, allowing the identification of functional groups and determination of the chemical structure of the tested adsorbent (interpolymer system). In order to verify the chemical structure of the Lewatit CNP LF and AV-17-8 ion exchangers which were subjected to sorption testing, as well as to observe the alterations induced by Eu(III) and Sc(III) ions, spectroscopic data for the initial ion exchangers were collected before/after the process of sorption involving the aforementioned ions. The graphical representations of the findings are depicted in the Results and Discussion section.

## 3. Results and Discussion

Effect of the Swelling Degree and Molar Ratios of Ion Exchangers in the Interpolymer Systems on the Degree of Europium and Scandium Ions Sorption

According to the experiments, the swelling degree of the Lewatit CNP LF in the interpolymer system was K_sw_ = 2.05, whereas this ion exchanger outside the interpolymer system exhibited a swelling degree of K_sw_ = 1.40. Similarly, the swelling degree of the AV-17-8 was K_sw_ = 2.23, while the swelling degree of this ion exchanger outside the interpolymer system was K_sw_ = 1.30. The polymer swelling implies a rise in permeability due to the hydration of exchange groups, which leads to the stretching of the polymer three-dimensional matrix and an increase in the sorption of europium and scandium ions.

The polymer chain binding degree was calculated according to Equation (2). The maximum obtained value of the polymer chain binding degree (*θ*) of europium ions was equal to 5.49% for the interpolymer system “Lewatit CNP LF:AV-17-8” (5:1) after 48 h of interaction (Table 1), whereas the polymer chain binding degree (*θ*) of scandium ions was equal to 19.33% for the interpolymer system “Lewatit CNP LF:AV-17-8” (2:4) after 48 h of interaction (Table 2).

**Figure 6:**
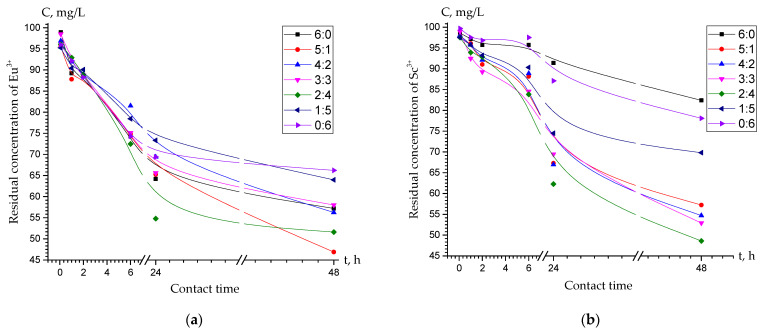
The residual concentration C of (**a**) europium and (**b**) scandium ions in a solution after sorption as a function of time.

The study revealed that the interpolymer system “Lewatit CNP LF:AV-17-8” (5:1) exhibited a 25% increase in sorption capacity for europium ions, compared to the raw Lewatit CNP LF (6:0). Similarly, the same interpolymer system showed a 57% growth in sorption capacity compared to the raw AV-17-8 (0:6) ionite, after 48 h of interaction (Figure 6a). Furthermore, the “Lewatit CNP LF:AV-17-8” (2:4) interpolymer system demonstrated a 310% increase in sorption capacity for scandium ions compared to the raw Lewatit CNP LF (6:0), whereas the same interpolymer system exhibited a 240% growth in sorption capacity compared to the raw AV-17-8 (0:6), after 48 h of interaction (Figure 6b).

**Figure 7:**
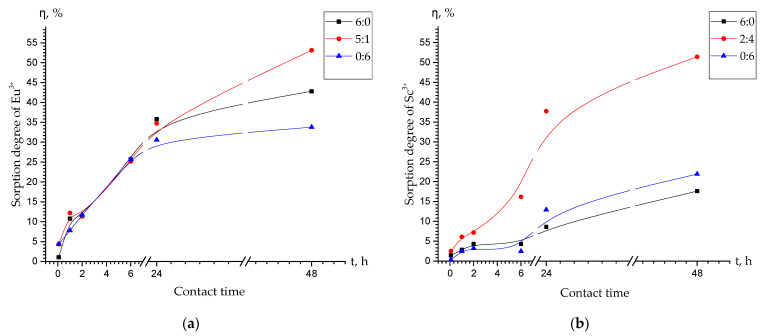
The dependence of the (**a**) europium and (**b**) scandium ions sorption degree as a function of time.

Figure 6 demonstrates, that the interpolymer systems Lewatit CNP LF:AV-17-8 (5:1) and Lewatit CNP LF:AV-17-8 (2:4) showed the highest sorption activity towards europium (Figure 6a) and scandium (Figure 6b) ions, respectively. As a result, the above-mentioned interpolymer systems were chosen for further detailed comparison with the raw Lewatit CNP LF (6:0) and raw AV-17-8 (0:6) ion exchangers (Figure 7). Figure 7a shows that after 48 h of interaction, the interpolymer system “Lewatit CNP LF:AV-17-8” (5:1) showed the maximum degree of europium ions sorption was equal to 53%, while raw Lewatit CNP LF (6:0) and raw AV-17-8 (0:6) showed 43% and 34%, respectively. On the other hand, Figure 7b represents that after 48 h of interaction, the interpolymer system “Lewatit CNP LF:AV-17-8” (2:4) showed the maximum degree of scandium ions sorption equal to 51%, whereas raw Lewatit CNP LF (6:0) and raw AV-17-8 (0:6) showed 21% and 16%, respectively.

**Figure 8:**
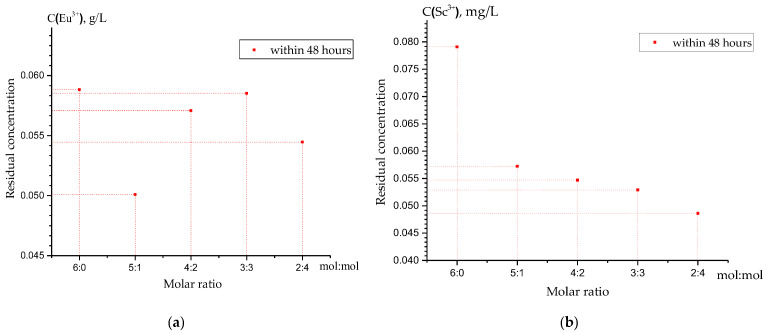
ICP-OES analysis of the residual concentration of (**a**) Eu^3+^ and (**b**) Sc^3+^ ions in a solution after 48 h of sorption.

The present study employed the ICP-OES analysis (Figure 8) to confirm the obtained data, where at a ratio of 5:1 (Figure 8a), the low values (the highest sorption) of the residual concentration of europium ions in the solution can be observed. At a ratio of 2:4 (Figure 8b), the low values (the highest sorption) of the residual concentration of scandium ions in the solution were also detected. The obtained experimental data confirm the enhanced sorption activity of the interpolymer systems “Lewatit CNP LF:AV-17-8” (5:1) and “Lewatit CNP LF:AV-17-8” (2:4) towards europium(III) and scandium(III) ions, respectively, from their nitrate solutions.

**Figure 9:**
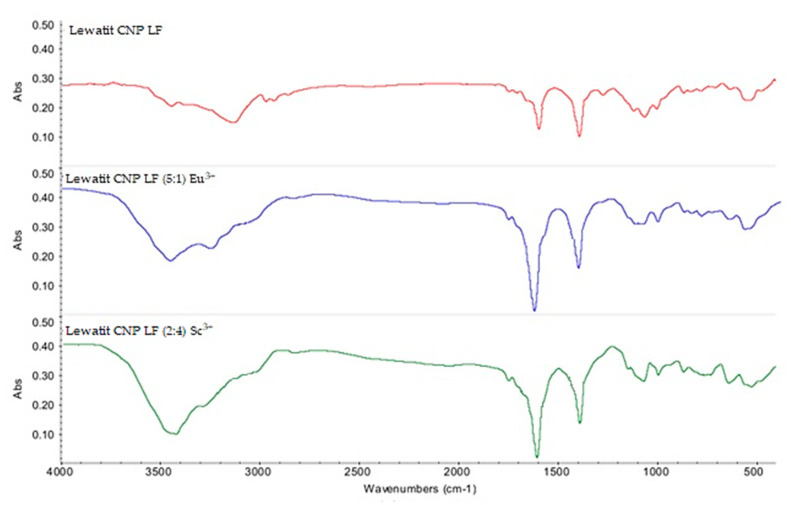
FTIR spectra: initial Lewatit CNP LF; Lewatit CNP LF (5:1) (with sorbed Eu^3+^) as part of the interpolymer system; Lewatit CNP LF (2:4) (with sorbed Sc^3+^) as part of the interpolymer system.

Figure 9 demonstrates the FTIR spectra of initial Lewatit CNP LF (without sorbed ions), Lewatit CNP LF (5:1) (with sorbed Eu^3+^ ions), and Lewatit CNP LF (2:4) (with sorbed Sc^3+^ ions). The changes in absorbance after the target REM sorption (wavenumbers interval 3300–2500 cm^−1^, which correspond to O-H stretching in the carboxylic group), wherein the values of absorbance in the case of the interpolymer systems disappeared, which, in turn, points to intense sorption of Eu^3+^ and Sc^3+^ ions by the interpolymer systems and possible replacement the hydrogen of the carboxylic group by sorbed metal ions.

**Figure 10:**
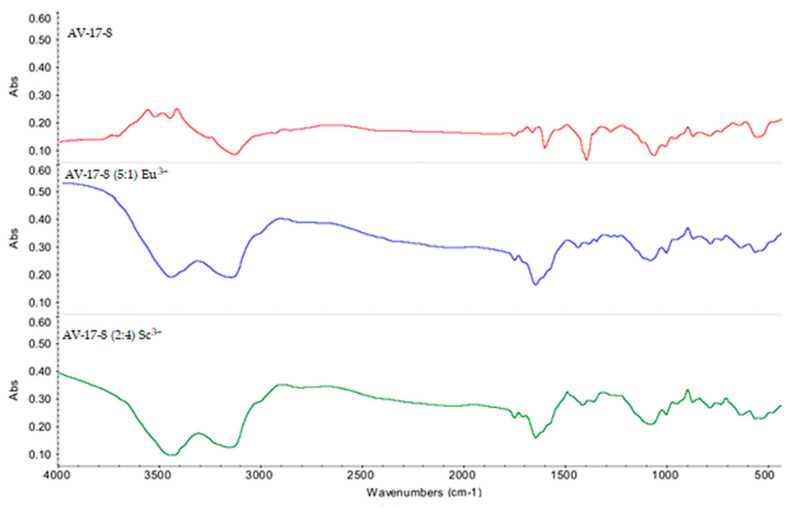
FTIR spectra: initial AV-17-8; AV-17-8 (5:1) (with sorbed Eu^3+^) as part of the interpolymer system; AV-17-8 (2:4) (with sorbed Sc^3+^) as part of the interpolymer system.

Figure 10 shows the FTIR spectra of initial AV-17-8 (without sorbed ions), AV-17-8 (with sorbed Eu^3+^ ions), and AV-17-8 (2:4) (with sorbed Sc^3+^ ions). Changes in absorbance after the target REMs sorption (wavenumbers interval 3400–3300 cm^−1^, which correspond to N-H stretching in the carboxylic group), wherein the values of absorbance in the case of the interpolymer systems disappeared, which, in turn, points to intense sorption of Eu^3+^ and Sc^3+^ ions by the interpolymer systems and possible replacement of the hydroxide group of the primary amine.

Fourier Transform Infrared (FTIR) spectroscopy, as a widely used analytical technique for the characterization of polymer sorbents, is a useful technique to provide detailed information on the chemical structure and composition of polymer sorbents and is particularly useful for identifying functional groups and chemical bonds within the polymer matrix. By analyzing the peaks and their intensities, we can gain insight into the chemical structure and composition of the polymer adsorbent and can use this information to optimize its performance for specific applications. This can include tailoring the polymer surface to enhance the sorption of specific types of metal ions, or to improve the overall efficiency of the sorption process.

## 4. Conclusions

The results obtained in this study demonstrate the potential application of interpolymer systems as efficient sorbents for the recovery of europium and scandium ions from solutions. The activation of polymers possessing acidic and basic characteristics by remote interaction in aqueous media has been shown to enhance their sorption activity due to the transition to a highly ionized state. Our investigation revealed that the interpolymer system “Lewatit CNP LF:AV-17-8” (5:1) exhibited a 25% increase in the degree of europium ion sorption compared to the raw Lewatit CNP LF (6:0), whereas the same interpolymer system displayed a 57% increase in the degree of europium ion sorption compared to the raw AV-17-8 (0:6) ion exchanger after a 48-h interaction period. Similarly, the interpolymer system “Lewatit CNP LF:AV-17-8” (2:4) exhibited a 310% increase in the degree of scandium ion sorption compared to the raw Lewatit CNP LF (6:0), and the same interpolymer system showed a 240% increase in the degree of scandium ion sorption compared to the raw AV-17-8 (0:6) after 48 h of interaction.

The increased sorption degree of europium and scandium ions by the interpolymer systems in comparison to the raw ion exchangers could be explained by the achievement of a high ionization state. The various degree of polyelectrolyte ionization caused by the remote interaction in aqueous media depends on the ratios of dissimilar ionic polymers and the interaction time. Thus, the remote interaction effect of functional polymers can provide a set of conformational states with a certain distribution of complementary structures to certain ionic radii of metals.

To further evaluate the potential for recovering europium and scandium ions from secondary sources and implementing the sorption process on an industrial level, future research may encompass the following investigations: (i) elucidating the mechanisms involved in the sorption/desorption processes and conducting economic analysis, (ii) exploring the regeneration of interpolymer systems, and (iii) expanding the scope of application studies. Such studies could offer valuable insights into the practical feasibility of europium and scandium ions recovery via sorption processes and inform potential industrial applications.

## Figures and Tables

**Table 1:** The polymer chain binding degree of the europium ions at different ratios of the ion exchangers (*θ*, %).

Time, h Ratio	0.08	1	2	6	24	48
6:0	0.11	1.11	1.17	2.65	3.69	4.41
5:1	0.45	1.26	1.17	2.60	3.59	5.49
4:2	0.32	0.84	1.20	1.92	3.17	4.53
3:3	0.15	0.76	1.17	2.59	3.57	4.36
2:4	0.41	0.74	1.15	2.87	4.71	5.04
1:5	0.50	1.00	1.04	2.25	2.79	3.77
0:6	0.42	0.82	1.23	2.69	3.20	3.54

**Table 2:** The polymer chain binding degree of the scandium ions at different ratios of the ion exchangers (*θ*, %).

Time, h Ratio	0.08	1	2	6	24	48
6:0	0.49	0.99	1.49	1.49	3.00	6.13
5:1	0.75	1.39	3.18	4.20	11.61	15.18
4:2	0.71	1.55	2.85	4.02	11.97	16.40
3:3	0.38	2.77	3.97	5.69	11.27	17.37
2:4	0.93	2.29	2.69	6.07	14.19	19.33
1:5	0.95	1.64	2.61	3.71	9.79	11.58
0:6	0.13	0.97	1.25	0.97	5.06	8.58

## Data Availability

The data presented in this study are available upon request from the corresponding author.
